# *Millettia speciosa* Champ., a Plant with Potential for Development: A Comprehensive Review of Botany, Phytochemistry, Health Benefits, and Applications

**DOI:** 10.3390/foods15132351

**Published:** 2026-07-02

**Authors:** Qingqing Huang, Kecheng Wu, Yang Yang, Enzheng Zhu, Xue Xiao, Shenghua Piao

**Affiliations:** 1 Traditional Chinese Medicine Research Institute, Guangdong Pharmaceutical University, Guangzhou 510006, China; qingqing.huang001@outlook.com (Q.H.); wkczy17@126.com (K.W.); 2112348121@gdpu.edu.cn (Y.Y.); zhuenzheng1511@163.com (E.Z.); 2 Guangdong Provincial TCM Key Laboratory for Metabolic Diseases, Guangdong Pharmaceutical University, Guangzhou 510006, China; 3 Institute of Analysis, Guangdong Academy of Sciences (China National Analytical Center Guangzhou), Guangzhou 510006, China

**Keywords:** *Millettia speciosa* Champ, chemical composition, pharmacological effects, safety, industrial applications

## Abstract

*Millettia speciosa* Champ. (*M. speciosa*) is a traditional medicinal and edible plant with notable nutritional value and diverse biological activities. Although previous studies have investigated its botanical characteristics, chemical composition, pharmacological effects, and potential applications, a systematic review remains lacking, limiting comprehensive understanding and further utilization of this plant. This review summarizes the botanical features and chemical constituents of *M. speciosa*, critically discusses its pharmacological activities, and evaluates its safety and beneficial health effects. While current experimental data suggest that *M. speciosa* has therapeutic potential, further validation is required, and significant research limitations remain, including the underutilization of its non-medicinal parts, the scarcity of clinical evidence, and limited progress in product development and commercial translation. These factors restrict further development and industrialization of this plant resource. Future studies should focus on several areas: the full utilization of whole-plant resources, the mechanisms of action of the plant’s active components, systematic toxicological evaluation and clinical translation, and the establishment of standardized quality evaluation systems. This may help unlock the full application potential of *M. speciosa*.

## 1. Introduction

Rising health awareness is driving market demand for natural and healthy products, with medicinal and edible plants being particularly valued for their combined nutritional and therapeutic benefits. *Millettia speciosa* Champ. (*M. speciosa*) is a traditional dual-use plant in China, commonly consumed in soups and teas for its tonic properties [1]. As a medicine, it exhibits effects such as relieving muscular stiffness and activating meridians, clearing heat and removing toxins, and nourishing deficiencies and moistening the lungs [2]. The plant is abundant in southern China, with stable and adequate supplies. Modern research has confirmed that *M. speciosa* contains polysaccharides, flavonoids, alkaloids, and other active ingredients [3]. These compounds not only constitute the material basis for its pharmacological effects, such as enhancing immunity, anti-fatigue, and anti-oxidant effects, but also provide a scientific basis for its traditional efficacy. As the health industry continues to develop, its application forms have gradually expanded from traditional soups and teas to a wider range of products, demonstrating promising development prospects.

Significant progress has recently been made in isolating, identifying, and pharmacologically evaluating the chemical constituents of *M. speciosa*, with most existing reviews focusing on its chemical composition and biological activities. However, a systematic review that integrates its resource characteristics, material properties, bioactivity, safety, and applications remains lacking.

This review, therefore, provides a broader and more multidimensional perspective. Proceeding from five dimensions, namely botany, phytochemistry, pharmacological effects, health benefits, and applications, and by analyzing the interconnections among them, this review systematically summarizes the research progress on *M. speciosa*, identifies current limitations, and proposes future directions. This work aims to elucidate the application potential of *M. speciosa* and to offer a theoretical and practical foundation for its further development and utilization.

## 2. Methodology

A systematic literature search was conducted using the electronic databases ScienceDirect, PubMed, and Web of Science, as well as the Chinese database China National Knowledge Infrastructure. This search was supplemented with information from Chinese herbal texts, including the Guangdong Chinese Materia Medica, Essentials of Raw Herbal Medicine, and Lingnan Herbal Records. The search period spanned from the inception of each database to 31 March 2026. Keywords included *Millettia speciosa* Champ., niudali, botany, chemical composition, pharmacological effects, toxicology, and applications. Eligible document types comprised peer-reviewed journal articles, reviews, and patents published in Chinese or English.

A total of 215 documents were initially retrieved. After removing duplicates using EndNote X9, the remaining records were screened stepwise based on the title, abstract, and full text. Through this process, 82 valid documents were finally included. From these, relevant data and conclusions on the botanical characteristics, types and contents of chemical constituents, pharmacological mechanisms, safety, and applications of *M. speciosa* were systematically extracted, categorized, and summarized. All chemical structures presented in this review were independently drawn by the authors using ChemDraw 21.0.0 software.

## 3. Botany and Distribution of *M. speciosa*

**Figure 1:**
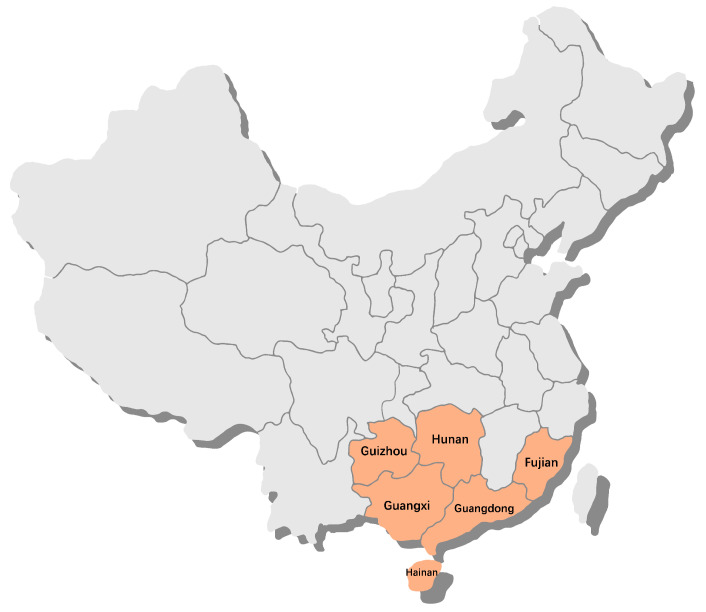
Distribution of *M. speciosa* in China.

*M. speciosa* belongs to the genus *Millettia* in the family Fabaceae and is a perennial woody vine. It is widely distributed in China, Indonesia, and Thailand. In China, it is mainly found in Guangdong, Guangxi, Hainan, Fujian, Hunan, Guizhou, and other provinces (Figure 1) [4]. It grows on mountain slopes, forest margins, and streamside areas at altitudes of 200 to 800 m. This distribution reflects its preference for warm, humid, and shaded environments and also indicates that it is abundant in southern China. Therefore, *M. speciosa* is known as southern medicine. However, due to its high medicinal value, wild *M. speciosa* was once over-excavated, resulting in a reduction in wild resources and highlighting the importance of its cultivation. Nowadays, cultivated *M. speciosa* has become the main source of market supply. It is worth noting that there is no fundamental difference in composition or effect between cultivated and wild *M. speciosa*. Metabolomics analysis via UHPLC-Q-Exactive Orbitrap-MS confirmed that the wild and cultivated products could be distinguished with a few specific markers. However, they were highly consistent in their overall chemical compositions, and the cultivated product retained significant biological activities [5]. This conclusion demonstrates that cultivated *M. speciosa*, produced through standardized planting, can effectively replace wild resources while maintaining quality and efficacy. This not only alleviates pressure on wild resources but also provides a resource guarantee for the future development and application of *M. speciosa*. For instance, five-year-old plants exhibited the highest flavonoid content in the roots and the most pronounced hepatoprotective effect. In contrast, roots from plants older than twenty years showed advantages in regulating metabolism and improving insulin resistance [6]. This study reveals the dynamic accumulation pattern of active ingredients in *M. speciosa*. Based on this characteristic, raw materials with suitable growth periods can be selected to meet the requirements of developing products.

**Figure 2:**
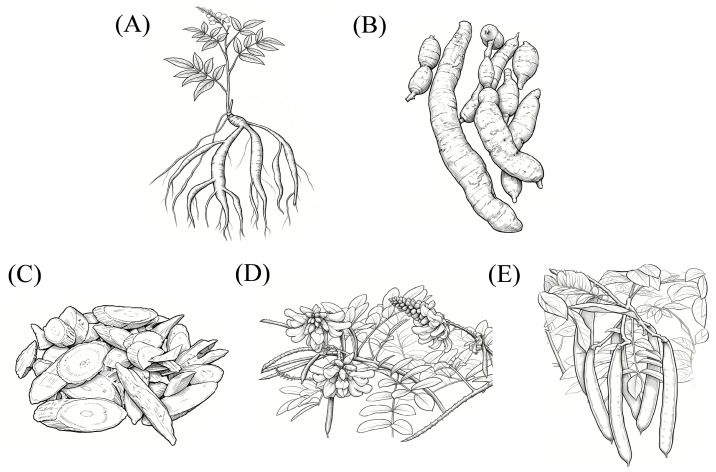
Different parts of *M. speciosa*: (**A**) whole plants; (**B**,**C**): roots; (**D**): stems, leaves, and flowers; (**E**): pods.

*M. speciosa* is an erect or twining subshrub that typically reaches 1–2 m in height (Figure 2A). The whole plant is usable, particularly the roots, which are cylindrical or composed of several fusiform segments in series, a structure that enables them to penetrate deeply into the soil (Figure 2B). This unique morphology provides the structural basis for nutrient storage [7], thereby establishing the root as the key site for active accumulation of constituents. Thus, the research indicated that *M. speciosa* roots contain various nutrients and dietary fibers, including cellulose, carbohydrates, proteins, vitamins, fats, amino acids, and mineral elements, at contents of 31%, 25%, 5%, 0.05%, 0.1%, 3.29%, and 2%, respectively [8]. Among these, carbohydrates and cellulose have the highest contents, suggesting that the roots are rich in saccharides or starch-like components. In addition, the roots are abundant in vitamins A (7.10 μg/g), B2 (40.92 μg/g), B3 (0.70 μg/g), B12 (16.02 μg/g), C (179.97 μg/g), and E (0.52 μg/g), among which vitamin C, known for its anti-oxidant activity, is present at a relatively higher level, indicating certain anti-oxidant potential. Furthermore, the roots contain the eight essential amino acids for humans: lysine, tryptophan, phenylalanine, methionine, threonine, isoleucine, leucine, and valine [8]. These amino acids play important roles in maintaining normal physiological functions and promoting healthy nutritional intake. Moreover, the plant’s roots are rich in various mineral elements, including calcium, magnesium, iron, strontium, aluminum, manganese, and zinc [9], which support bone health and immune regulation. This botanical feature not only confirms the application value and importance of the root but also provides a clear direction for subsequent research on chemical components and product development. In practice, the roots are usually sliced into thin pieces to maximize their efficacy (Figure 2C). In addition to the root, *M. speciosa* also has stems, leaves, flowers (Figure 2D), pods (Figure 2E) and other parts. While current research has primarily focused on the roots, explorations targeting these aerial parts have progressively increased in recent years. Traditionally, only the roots of *M. speciosa* were used, which led to wasted plant resources. However, modern research has revealed that the aboveground parts also possess significant value. For example, the stem contains active ingredients similar to those found in the root, along with unique chemical substances [10], making it suitable as a raw material for characteristic extraction. The leaf is rich in protein that, when extracted, has high nutritional value and exhibits good emulsifying and foaming properties [11]. The flower is abundant in volatile components, while the seeds are notable for their high unsaturated fatty acid content. These findings gradually reveal the comprehensive utilization potential of the whole *M. speciosa* plant.

In summary, the botanical characterization of *M. speciosa* has confirmed its value for application from multiple perspectives. However, botanical research on the stems, leaves, flowers, and other parts remains insufficient. This knowledge gap hinders the study of the chemical composition and biological activity of these parts, thereby limiting the comprehensive utilization of the whole plant. Addressing this gap represents a key direction for future research and development, which is essential for realizing the full potential of this plant.

## 4. Phytochemistry of *M. speciosa*

An analysis of *M. speciosa*’s botanical characteristics reveals that its roots, stems, leaves, and other parts all possess research value. To elucidate the plant’s chemical composition, researchers conducted a systematic chemical analysis. To date, researchers have isolated and identified 61 compounds from the plant, which can be classified into eight major groups: polysaccharides, flavonoids, alkaloids, organic acids, triterpenoids, sterols, coumarins, and lignans (Table 1) [3]. These abundant chemical constituents account for the plant’s wide range of pharmacological activities.

The *M. speciosa*’s roots are rich in polysaccharides, which exhibit diverse bioactivities, notably including enhancing immunity, anti-fatigue, anti-oxidation, anti-inflammation, hepatoprotective, cough-relieving, and hypoglycemic effects [12]. Studies have demonstrated that the biological activities of its polysaccharides are closely associated with structural characteristics, including molecular weight, monosaccharide composition, and glycosidic bond types. Rongrong Cheng et al. used infrared spectroscopy combined with ion chromatography to confirm that the water-soluble polysaccharide MSP-1 is composed of rhamnose, galactose, glucose, mannose, and fructose [13]. Additionally, Huang et al. conducted structural characterization of the novel polysaccharide MSCP2. They found that it is composed of fucose, arabinose, galactose, glucose, and xylose, and also identified α-D-Glcp-(1 → 4), α-D-Glcp-(1→), α-D-Xylp-(1 → 6), β-D-Galp-(1→), α-L-Araf-(1 → 3,4), β-L-Fucp-(1→), and →4)-α-D-GalpA-(1→ as its main glycosidic bond type [14]. The difference in monosaccharide composition between these two polysaccharides suggests that they may exhibit distinct biological activities, providing a direction for deepening the exploration and application of the pharmacological functions of *M. speciosa* polysaccharides.

Flavonoids are one of its core active ingredients, and 30 different flavonoid compounds, mainly belonging to chalcones and isoflavones, have been isolated and identified from roots, stems and other parts of the plant. Activity experiments indicated that *M. speciosa* flavonoids exhibit anti-inflammatory, anti-oxidative, anti-fatigue, and hepatoprotective effects [3]. Among these components, formononetin and maackiain are the primary chemical components of *M. speciosa*, whose levels are closely linked to the plant’s quality and thus are frequently used as marker substances to assess the quality [15]. It is worth noting that most studies on bioactivity have been confined to crude extracts. Although formononetin and maackiain have been employed as quality markers, the direct link between their content and specific pharmacological activity remains unclear.

*M. speciosa* also contains alkaloids, an important class of bioactive compounds in plants. Alkaloids generally possess neuroprotective and cardioprotective activities. To date, only 4 alkaloids have been isolated from this plant, and investigations have been limited to evaluating their anti-oxidant activity [16]. Consequently, the pharmacological potential of these alkaloids warrants further investigation. Furthermore, an integrated quality marker screening strategy combining chromatographic fingerprint analysis, serum pharmacochemistry, network pharmacology, and quantitative analysis has been successfully established [17]. This approach identified the alkaloid compound lenticin as a quality marker of *M. speciosa*, providing a scientific basis for the quality control and standardization of this plant.

Although organic acids, triterpenoids, sterols, coumarins, and lignans have been identified in *M. speciosa*, research on these compounds remains at the preliminary analytical stage, and little is known about their specific biological activities.

**Table 1:** The chemical components of *M. speciosa*.

Classification of Compounds	Active Effect	Name of Components	Source Part	Extraction	Formula	Molecular Weight (g/mol)	Structures	Ref.
Polysaccharides	Enhancing immunity Anti-fatigue Anti-oxidation Anti-inflammation Hepatoprotective Cough-relieving Hypoglycemic effects	MSP-1	Roots	Aqueous	\	\	\	[13]
		MSCP2			\	2.85 × 10^4^	\	[14]
Flavonoids	Enhancing immunity Anti-inflammatory Anti-oxidative Anti-fatigue Hepatoprotective effects	3,4,2′,4′-Tetrahydroxy chalcone	Roots and stems	Ethanol	C_15_H_12_O_5_	272.25	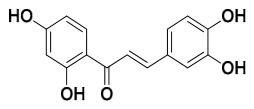	[18]
		Isoliquiritigenin			C_15_H_12_O_4_	256.25	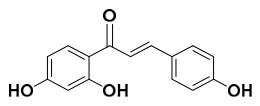	[19]
		2′,4,4′,α-Tetrahydroxydihydrochalcone	Roots		C_15_H_14_O_5_	274.27	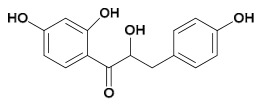	[18]
		4-Hydroxy-2′,4′-dimethoxychalcone			C_17_H_16_O_4_	284.31	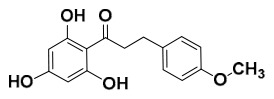	
		2′,4′,α-Trihydroxy-4-methoxydihydrochalcone			C_16_H_16_O_5_	288.3	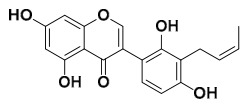	
		2′-Hydroxybiochanin A			C_16_H_12_O_6_	300.26	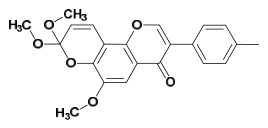	
		2′,5′,7-Trihydroxy-4′-methoxyisoflavone			C_16_H_12_O_6_	300.26	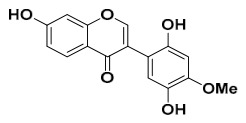	
Flavonoids	Enhancing immunity Anti-inflammatory Anti-oxidative Anti-fatigue Hepatoprotective effects	7-Hydroxy-6,4′-dimethoxyisoflavone	Roots	Ethanol	C_17_H_14_O_5_	298.29	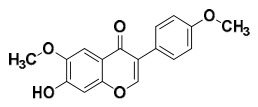	[20]
		Psi-Baptigenin			C_16_H_10_O_5_	282.25	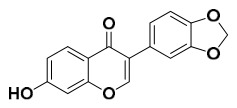	
		Maackiain			C_16_H_12_O_5_	284.26	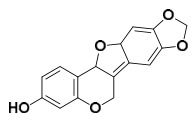	
		Licochalcone A			C_21_H_22_O_4_	338.39	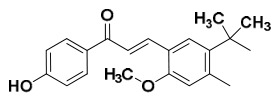	
		3′,4,7-Trihydroxyisoflavone			C_15_H_10_O_5_	270.24	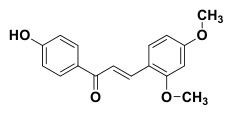	[21]
		Licoisoflavone A			C_20_H_18_O_6_	354.35	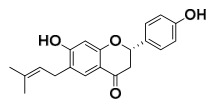	
		Echinatin			C_16_H_14_O_4_	270.28	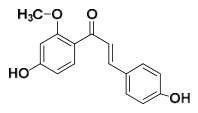	
		3′,7-Dihydroxy-2,4′-Dimethoxyisoflavone			C_17_H_14_O_6_	314.28	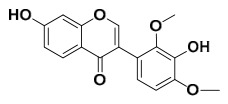	
		Formononetin			C_16_H_12_O_4_	268.26	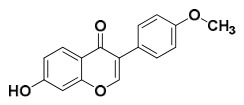	[22]
		Homopterocarpin			C_17_H_16_O_4_	284.3	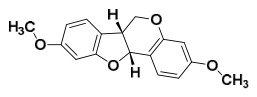	
		Medicarpin			C_16_H_14_O_4_	270.28	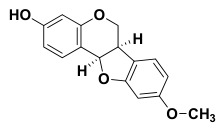	
		Quercetin			C_15_H_10_O_7_	302.23	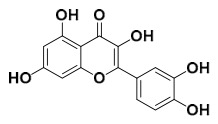	
Flavonoids	Enhancing immunity Anti-inflammatory Anti-oxidative Anti-fatigue Hepatoprotective effects	Isoquercitrin	Roots	Ethanol	C_21_H_20_O_12_	464.37	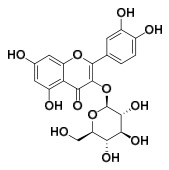	[22]
		Calycosin			C_16_H_12_O_5_	284.26	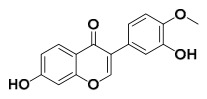	[23]
		Iristectorigenin A			C_17_H_14_O_7_	330.28	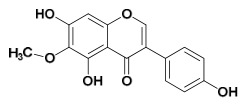	
		6-Methoxycalopogonium isoflavone A			C_22_H_20_O_6_	380.4	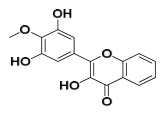	
		7-Hydroxy-6,4′-dimethoxyisoflavone			C_17_H_14_O_5_	298.29	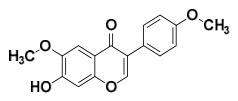	
		Pterocarpin			C_17_H_14_O_5_	298.29	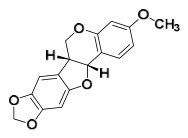	[24]
		Amentoflavone			C_30_H_18_O_10_	538.45	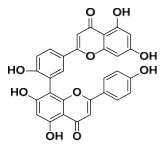	
		Sulfurein			C_15_H_10_O_5_	270.23	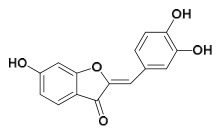	
		Liquiritigenin			C_15_H_12_O_4_	256.25	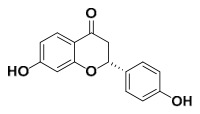	
		Naringenin			C_15_H_12_O_5_	272.25	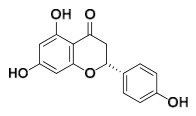	
		4′-Hydroxy-7-methoxyflavan	Stems		C_16_H_14_O_4_	270.28	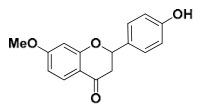	[18]
Alkaloids	Anti-oxidation	6-Methoxydihydrosanguinarine	Roots	Ethanol	C_21_H_17_NO_5_	363.36	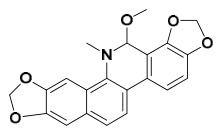	[19]
		N-Methylcytisine			C_12_H_16_N_2_O	204.27	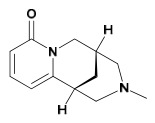	
		Aurantiamide acetate			C_27_H_28_N_2_O_4_	444.52	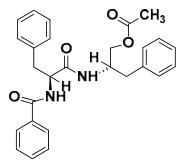	
		β-Erythroidine			C_16_H_19_NO_3_	273.32	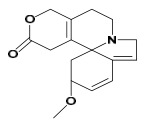	[25]
Organic acids	\	2,5-Dihydroxybenzoic acid	Roots	Ethanol	C_7_H_6_O_4_	154.12	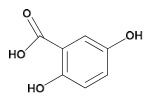	[18]
		Vanillic acid			C_8_H_8_O_4_	168.14	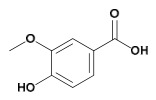	[19]
		Syringic acid			C_9_H_10_O_5_	198.17	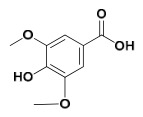	
		Hexacosanoic acid			C_26_H_52_O_2_	396.69	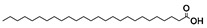	
		Maleic acid			C_4_H_4_O_4_	116.07	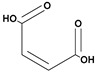	
		Docosanoic acid			C_22_H_44_O_2_	340.58	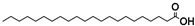	[26]
		Linoleic acid			C_18_H_32_O_2_	280.44	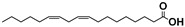	
Triterpenoids	\	Shionone	Roots	Ethanol	C_30_H_50_O	426.71	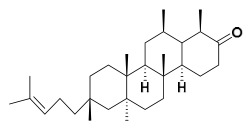	[19]
		Lupeol caffeate			C_39_H_56_O_4_	588.86	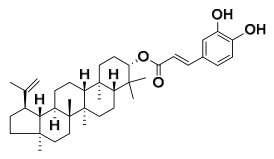	
		Glycyrrhizic acid			C_42_H_62_O_16_	822.93	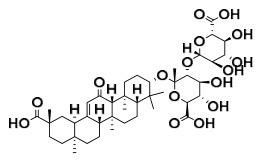	
		7-β-hydroxylathyrol			C_20_H_32_O_2_	350.44	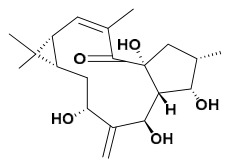	
		Pyracrenic acid			C_39_H_54_O_6_	618.84	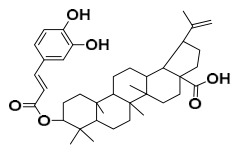	[21]
		Rutundic acid			C_30_H_48_O_5_	488.69	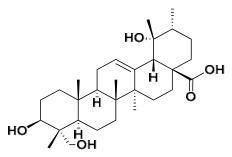	
		Pedunculoside			C_36_H_58_O_10_	650.84	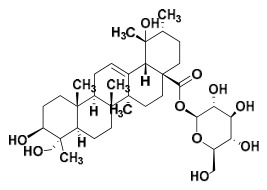	
Sterols	\	7-Oxo-β-sitosterol	Roots	Ethanol	C_30_H_50_O_2_	442.73	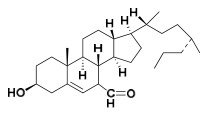	[19]
		Stigmasterol	Stems	Ethanol	C_29_H_48_O	412.69	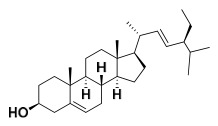	[21]
		β-Sitosterol			C_29_H_50_O	414.7	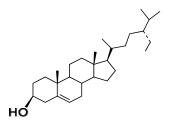	
		Stigmasterol-3-O-β-D-glucopyranoside			C_35_H_50_O_6_	574.83	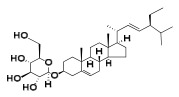	
		β-Daucosterol			C_35_H_60_O_6_	576.84	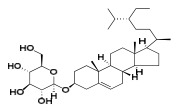	
		β-Sitosterol acetate			C_31_H_52_O_2_	456.74	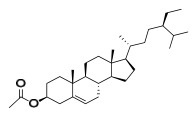	
Coumarins	\	Psoralen	Roots	Ethanol	C_11_H_6_O_3_	186.16	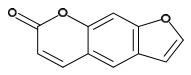	[19]
Lignins	\	Syringaresinol	Roots	Ethanol	C_22_H_26_O_8_	418.43	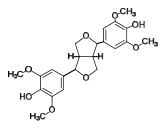	[19]
		Schisandrol B			C_23_H_28_O_6_	400.46	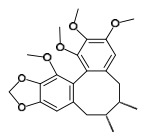	
		Secoisolariciresinol			C_20_H_26_O_6_	362.41	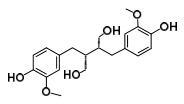	[21]
		Dihydrodehydrodiconiferyl alcohol			C_20_H_24_O_6_	360.4	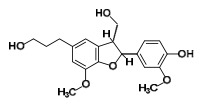	

In summary, a variety of chemical constituents have been isolated and identified from *M. speciosa*, and the pharmacological activities of components such as polysaccharides and flavonoids have been preliminarily confirmed. However, current research still has significant shortcomings. On the one hand, bioactivity studies have largely remained at the level of crude extracts, and the identified individual compounds have not been evaluated for bioactivity, leaving the pharmacological basis unclear. On the other hand, the biological activity of chemical constituents is closely related to their structural characteristics; nevertheless, systematic structure–activity relationship studies have not yet been conducted, and the intrinsic patterns linking structural features to biological activity remain to be explored. For example, the two polysaccharides isolated from *M. speciosa* exhibit distinct monosaccharide compositions and glycosidic bond configurations. Yet, no studies have reported whether there are differences in their pharmacological activities or potencies. These limitations suggest that comprehensive investigations into the biological activities of the various chemical constituents of *M. speciosa* are a necessary prerequisite for fully evaluating its application potential.

## 5. Pharmacological Effects of *M. speciosa*

**Figure 3:**
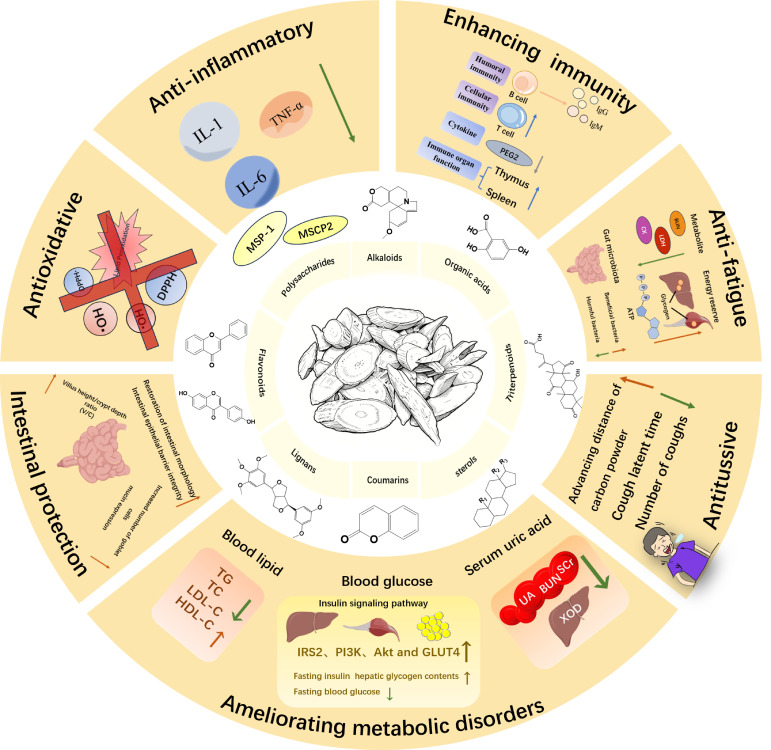
The phytochemistry and pharmacological effects of *M. speciosa*. Note: ↑ means increased, and ↓ means decreased.

Modern studies have confirmed that *M. speciosa* exhibits a variety of pharmacological effects, including enhancing immunity, anti-fatigue, anti-oxidant, anti-inflammatory, antitussive, hypoglycemic, and uric acid-lowering activities [27]. Table 2 summarizes the main pharmacological effects and their corresponding active components, while Figure 3 illustrates the plant’s phytochemistry and pharmacological effects. However, existing studies vary considerably in their subjects, experimental models, and dosing regimens.

Specifically, existing studies have not adopted standardized methods for subject selection. Extraction methods primarily include aqueous and ethanol extraction, yielding aqueous and ethanol extracts, respectively, along with polysaccharides and flavonoids. Aqueous extracts are rich in polysaccharides and polar compounds, whereas ethanol extracts are enriched with flavonoids, alkaloids, and other moderately polar compounds. Polysaccharides and flavonoids, in contrast, are isolated as purified single-component fractions. Although most pharmacological studies use the same type of extract, differences in extraction processes lead to variations in active ingredient content. This variation may explain the high dosage levels in some studies. Thus, the chemical constituents vary markedly across studies, further complicating comparisons.

Furthermore, the models used by different research institutions vary, even when studying the same pharmacological effects. This variation often reflects different research objectives. This enables a comprehensive assessment of *M. speciosa*’s pharmacological effects. This suggests that its activity may involve multiple pathways or regulatory mechanisms operating at different levels to achieve the same outcome. Take enhancing immunity as an example. Cellular experiments help elucidate the underlying molecular mechanisms, while animal studies demonstrate the overall improvement in immune function, reflecting the compound’s actual efficacy. These two approaches complement each other at the molecular and systemic levels, forming a comprehensive chain of evidence. Animal experiments show that *M. speciosa* positively modulates both pathological and normal physiological states, with efficacy observed after both short and long-term administration. This provides a clear direction for developing therapeutic and daily health products.

Traditionally, *M. speciosa* has been used to treat pleurisy, chronic hepatitis, and other inflammation-related conditions. Modern research has confirmed that this effect is primarily due to its polysaccharides and flavonoids. In vitro anti-oxidant activity assays have shown that both the polysaccharides and flavonoids from *M. speciosa* exhibit potent free radical scavenging capabilities [13,28]. Regarding the anti-inflammatory mechanism, cellular and animal studies have demonstrated that the polysaccharides primarily alleviate inflammation by inhibiting the NF-κB signaling pathway, thereby reducing the release of inflammatory cytokines such as IL-1, IL-6, and TNF-α. Recent studies have shown that the low molecular weight oligosaccharides from *M. speciosa* exhibit stronger anti-inflammatory activity than its high molecular weight polysaccharides [29]. This finding suggests that molecular weight may be a key structural factor influencing this activity and provides a direction for developing products with potent anti-inflammatory effects. In addition, animal studies have shown that flavonoids downregulate IL-6 and TNF-α levels in the lungs, demonstrating a clear therapeutic effect against pulmonary inflammation [30]. In summary, these findings provide preliminary insights into the biological mechanisms of its potential application in inflammation-related diseases.

Enhancing immunity is a key manifestation of *M. speciosa*’s “tonifying deficiency” effect. Animal studies have shown that its extract regulates immune organ function, immune cell activity, and antibody levels, with polysaccharides serving as the key active compounds. These polysaccharides promote the proliferation of antibody-producing cells, modulate T-lymphocyte function, and enhance immunity by activating the TLR4/MyD88/NF-κB signaling pathway, which stimulates macrophages to release immune factors [31].

*M. speciosa* is also used to relax muscles and tendons, as well as to tonify deficiency and boost vitality. Modern animal models of fatigue have confirmed its anti-fatigue effects, which are primarily achieved through the regulation of energy metabolism. This includes increasing liver glycogen stores and reducing fatigue-related markers such as blood urea nitrogen and lactate. Recent studies have also shown that *M. speciosa* modulates the gut microbiota in mice, increasing the abundance of beneficial bacteria. This improvement in gut microecology further enhances exercise endurance and alleviates fatigue [32].

An animal study has shown that *M. speciosa* has antitussive effects. Its aqueous extract significantly improved outcomes such as prolonged cough latency and reduced cough frequency, with effects comparable to those of the positive control drug dextromethorphan. This finding supports the traditional use of *M. speciosa* for cough relief [33]. In addition, its traditional use for strengthening muscles and bones has been supported by modern cellular studies. Triterpenoids and sterols isolated from its roots, such as lupeol and β-sitosterol, have been shown to inhibit osteoclast formation [34]. This indicates that these compounds could be valuable for preventing and treating lumbar muscle strain and other bone diseases.

In recent years, *M. speciosa* has shown new activity in the treatment of metabolic disorders. Its extract significantly reduces serum uric acid levels and alleviates associated kidney damage in rats with hyperuricemia [35]. Related animal studies suggest that it also lowers blood glucose and blood lipids, enhances insulin sensitivity, and regulates hepatic lipid metabolism. These effects are closely linked to its flavonoids and polysaccharides. Flavonoids show greater potential for lowering blood glucose, regulating blood lipids, and combating obesity, with formononetin exhibiting notable anti-metabolic syndrome activity [36]. Polysaccharides, conversely, promote insulin secretion and activate insulin signaling pathways, thereby exerting hypoglycemic effects [37].

In terms of intestinal protection, polysaccharides from *M. speciosa* upregulate the expression of intestinal barrier-related proteins, repair both the physical and chemical barriers of the intestine, and reshape the gut microbiota by increasing beneficial metabolites such as acetate and butyrate. Integrated metabolomic and network pharmacology analyses have also revealed antidepressant activity of *M. speciosa*. It reverses metabolic disturbances in the serum and brain of mice with chronic stress-induced depression and modulates pathways involved in tryptophan metabolism and neurotransmitter synthesis [38]. Urinary metabolomics in rats with chronic unpredictable mild stress-induced depression further showed that *M. speciosa* exerts antidepressant effects by regulating multiple metabolic pathways, including those of branched-chain amino acids, tyrosine, and histidine. L-Isoleucine, sebacic acid, and allantoin were identified as potential pharmacodynamic biomarkers [39]. Additionally, *M. speciosa* exhibits significant activity in protecting the reproductive system. It improves sperm quality, restores testosterone levels, and protects and repairs the blood-testis barrier in mice with testicular injury [40].

The studies reviewed above confirm the broad pharmacological activity of *M. speciosa* from multiple perspectives, while also revealing certain limitations in current research. At present, the evaluation indicators for the pharmacological effects of *M. speciosa* are diverse but lack standardized criteria. Existing studies primarily rely on classical endpoint indicators such as organ indices, serum biochemical parameters, cytokine levels, and anti-oxidant enzyme activities. Although these methods reflect overall physiological effects, they are insufficient for causal validation of molecular targets and signaling pathways. Furthermore, the inconsistent use of positive controls across studies further hinders the assessment of its efficacy. To address these issues, it is recommended that BUN, liver glycogen, and muscle glycogen be used as core indicators when evaluating anti-fatigue effects. For evaluating immune-enhancing effects, immune organ indices, immune cell counts, and immunoglobulin levels should be adopted as primary indicators. In addition to the lack of standardized evaluation indicators, the mechanisms of action remain insufficiently explored. Most studies have been limited to animal models and lack validation of specific molecular targets or support from multi-omics data. Consequently, the precise mechanisms underlying its diverse pharmacological effects remain unclear. Future studies should integrate transcriptomics, metabolomics, and network pharmacology to construct a multi-target network of action, thereby comprehensively elucidating its mechanisms.

**Table 2:** The pharmacological effects of *M. speciosa*.

Pharmacological Effect	Extract or Compound	Study Design	Model	Dose	Time of Treatment	Positive Control Drug	Result/Mechanism	Ref.
Enhancing immunity	Aqueous extract from *M. speciosa* roots	In vivo	NIH mice	30, 40, 50 g/kg d	15 days	/	Number of hemolytic plaques produced by B lymphocytes and the effect of serum anti-SRBC antibody ↑	[41]
			SD rat (cyclophosphamide-induced)	0.1, 0.2, 0.4 g/kg	10 days	/	Levels of serum IgG and IgM ↑	[42]
			KM mice	0.15, 0.3, 0.6 g/kg	15 days	Vitamin C	Spleen index, thymus index, phagocytic index α, number of hemolytic plaques ↑	[43]
			Immunosuppressed KM mice (prednisone acetate tablets-induced)	5, 10, 20 g/kg d	12 days	/	Spleen index, thymus index, and clearance index ↑	[44]
	Ethanol extract from *M. speciosa* roots		KM mice (cyclophosphamide-induced)	5, 10, 20 mg/kg	20 days	/	Body weight of mice, index of immune organs, number of WBC, degree of delayed-type hypersensitivity, phagocytic function of macrophages, spleen index, and thymus index ↑	[45]
	Polysaccharides		NIH mice (cyclophosphamide and tumor-bearing-induced)	100, 200, 400 mg/kg	10 days	/	IgM antibody, the number of antibody-forming cells, the phagocytic ability of macrophages, the proliferation and secretion of B lymphocytes ↑	[31]
	Aqueous extract from *M. speciosa* roots	in vitro	Spleen cell	100 μL	48 h	/	Levels of serum IgG and IgM ↑	[42]
	Polysaccharides		T lymphocyte	200 μg/mL	48 h	/	The proliferation of T lymphocytes, TNF-α, IL-6 ↑ PGE2 ↓	[46]
			T lymphocyte	4, 20 g/mL 5, 25, 50, 125, 160, 800 μg/mL	48 h	/	Proliferation of T lymphocytes in 5, 25, 50, 125 μg/mL ↑ Proliferation of T lymphocytes in 160, 800 μg/mL, 4, 20 g/mL ↓	[47]
Anti-fatigue	Aqueous extract from *M. speciosa* roots	In vivo	KM mice	5, 10, 20 g/kg	14 days	/	Swimming duration of mice ↑	[48]
			Sub-Health KM mice	20, 40, 80 g/kg	9 days	/	Exhaustive swimming time of mice ↑ BUN ↓	[49]
	Ethanol extract from *M. speciosa* roots							
	Polysaccharides		KM mice	212.5, 425, 850 mg/kg·d	15 days	Ginseng Royal Jelly	Time of mice climbing the rod and swimming with load, LDH ↑ LA and BUN↓	[50]
			ICR mice	200, 400, 800 mg/kg	30 days	Taurine	Climbing pole time and swimming time of mice, levels of muscle glycogen, liver glycogen, blood glucose, adenosine triphosphate, glutathione peroxidase, and superoxide dismutase, beneficial bacteria (Lactobacillus, Alistipes, Ruminococcaceae, and Roseburia) ↑ Levels of LDH, CK and BUN, harmful bacteria (Helicobacter, Anaerotruncus, Erysipelatoclostridium) ↓	[32]
Anti-oxidant	Aqueous extract, ethanol precipitate, and polysaccharides from *M. speciosa* roots	in vitro	·OH	0.15 mol/L	60 min	Vitamin C	Anti-lipid peroxidation, ability of scavenging ·OH free radicals and DPPH· free radicals ↑	[13]
	Total flavonoids		·OH	/	30 min		Ability of scavenging ·OH free radicals ↑	[28]
			DPPH·	/	60 min			
Anti-inflammatory	Aqueous extract from *M. speciosa* roots	in vivo	KM mice (xylene-induced)	5, 10, 20 g/kg d	4 days	Indometacin	Mouse ear swelling degree ↓	[51]
			SD rat (cotton pellet-induced)	2.5, 5, 10 g/kg d	10 days		granuloma inhibition rate ↓	
			KM mice (xylene-induced)	0.01 mL/g	7 days	Hydrocortisonedexamethasone	Mouse auricle swelling inhibition rate ↑	[52]
	Total flavonoids		Acute lung injury KM mice (LPS-induced)	10, 20, 40 mg/kg	7 days	Dexamethasone acetate	Number of WBC in bronchoalveolar lavage fluid, protein exudation volume, protein level of NF-κB p65 in lung tissue, mRNA expression of IL-6 and TNF-α, and levels of IL-6 and TNF-α in lung tissue ↓	[30]
	Polysaccharides							
		in vitro	RAW 264.7 cells (LPS-induced)	10, 100, 1000 ng/mL	12 h	/	Expression of IκB-α protein ↑ Release of inflammatory cytokines IL-1, IL-6, and TNF-α ↓	[53]
Hepatoprotective effect	Aqueous extract from *M. speciosa* roots	in vivo	KM mice (CCl_4_-induced)	5, 10, 20 g/kg d	12 days	Bifendate	Activities of AST and ALT in the serum, the content of MDA in the liver homogenate, liver index ↓	[54]
			Hepatic fibrosis zebrafish (diethylnitrosamine-induced)	25, 50, 100 mg/L	7 days	/	Expression levels of α-SMA, TNF-α**,** Bax, Collagen-1 ↓	[55]
	Ethanol extract from *M. speciosa* roots		Hepatic fibrosis mice (CCl_4_-induced)	3.5, 7, 14 g/kg	28 days	Colchicine	Level of SOD↑ Concentration of ALT, AST, MDA, Level of LN, HA, PCIII, IV-C, TGF-β1, IL-1β, IL-8, IL-6 ↓	[56]
	Polysaccharides		KM mice (CCl_4_-induced)	0.05, 0.1, 0.2 g/kg	8 days	Bifendate	Activities of ALT, AST in serum, level of MAD, liver index, expression levels of COX-2 ↓	[57]
Expectorant effect	Aqueous extract from *M. speciosa* roots	in vivo	Pigeon (ciliary motion)	4, 8, 16 g/kg d	3 days	Mucosolvan	Advancing distance of carbon powder ↑	[33]
Antitussive effect	Aqueous extract from *M. speciosa* roots	in vivo	Cough KM mice (ammonia water-induced)	5, 10, 20.g/kg d	5 days	Dextromethorphan	Cough latent time, number of coughs ↓	
Anti-asthmatic effect	Aqueous extract from *M. speciosa* roots	in vivo	Cavy (acetylcholine chloride-induced)	4, 8, 16 g/kg d	7 days	Aminophylline	Cough latent time, number of coughs ↓	[33]
Hypoglycemic effect	Polysaccharides	in vivo	Diabetic KM mice (STZ-induced)	100, 200, 400 mg/kg	28 days	Metformin Hydrochloride	Fasting insulin, hepatic glycogen contents ↑ Fasting blood glucose ↓	[37]
	Ethanol extract from *M. speciosa* roots		Diabetic KM mice (STZ-induced)	4.55, 9.10, 13.65 mg/kg d	10 weeks	Metformin Hydrochloride	Level of blood glucose, sensitivity of insulin, serum TC, TG, LDL-C ↓ Weight of diabetic mice, serum HDL-C, IRS2, PI3K, Akt and GLUT4 in liver, adipose and muscle tissues ↑ Improved the liver and pancreas tissue morphology	[36]
Reduce the level of uric acid	Aqueous extract from *M. speciosa* roots	in vivo	Uric acid nephropathy rat (potassium oxonate-induced)	2.3, 4.6, 9.2 g/kg	14 days	Allopurinol	Contents of UA, BUN, and SCr in the blood, activity of XOD in the liver ↓	[35]
Intestinal protection	Polysaccharides	in vivo	KM mice (cyclophosphamide-induced)	100, 200, 400 mg/kg d	14 days	Astragalus polysaccharides	Body weight, immune organ indices, the secretion of immune-related cytokines (IL-2, IL-4, IL-10, TNF-a, and IgG) ↑ Restoring intestinal morphology, the ratio of villus height/crypt depth (V/C), the number of goblet cells and mucins expression ↑	[38]
			Colitis C57BL/6 mice (dextran sulphate sodium-induced)	50, 100 mg/kg d	44 days	/	Production of anti-inflammatory cytokines, integrity of intestinal epithelial barrier ↑ Expression of TLR4, secretion of pro-inflammatory cytokines ↓	[58]
Antidepressant effect	Aqueous extract from *M. speciosa* roots	in vivo	KM mice with CUMS	20.0 g/kg d	35 days	fluoxetine	The ethology of depression (including sucrose preference degree, crossing lattice numbers and stand-up times), BDNF ↓ NE and 5-HT ↑ Improve depression through synergistically regulating five targets including Maoa, Maob, Ache, Ido1 and Comt, and three metabolic pathways such as tryptophan metabolism, synthesis of neurotransmitter and phospholipid metabolism	[59]
			SD rat with CUMS	3.5, 7, 14 g/kg d	42 days		Body weight, sucrose preference degree ↓ Urine metabolic showed that the profiles of the CUMS model group were significantly separated from the control group, while the drug-treated groups were closer to the control group	[36]
Protection of the reproductive system	Aqueous extract from *M. speciosa* roots	in vivo	ICR mice with testicular dysfunction (administered cyclophosphamide)	400, 800 mg/kg d	4 weeks	/	Body weight, testicular index, and epididymal index ↓ SOD, GSH-Px, upregulated related genes (Sod1, Sod2, Sod3, and Cat), sperm quality ↑	[40]

Note: ↑ means increased, and ↓ means decreased.

Despite the methodological and mechanistic limitations discussed above, *M. speciosa*, as a medicinal and edible plant, has had its traditional efficacies well validated by long-term folk practice. Building on its application in humans, subsequent animal studies have further clarified its underlying pharmacological mechanisms. Given the alignment between traditional experience and pharmacological evidence, animal studies on *M. speciosa* show potential for clinical translation, providing a solid scientific foundation for its application and development.

## 6. Safety and Health Benefits of *M. speciosa*

Due to its rich chemical composition and clear pharmacological mechanisms, *M. speciosa* is a medicinal and edible plant native to southern China. As one of its primary production regions, Guangdong Province has issued a local food safety standard [60] for *M. speciosa*, which specifies a recommended daily intake of ≤8 g for its dried root product. The standard provides a regulatory foundation for the further development of *M. speciosa* as a functional food ingredient. On this basis, *M. speciosa* holds broad application prospects.

From a safety perspective, several toxicological studies have fully confirmed the safety of *M. speciosa*. According to China’s “Procedures for Toxicological Evaluation of Food Safety” [61] , a substance should not be used in food if its LD_50_ is less than 100 times the recommended limit for human intake. Acute toxicity tests on *M. speciosa* root extract have shown that a single oral dose of 20.0 g/kg body weight did not cause any significant toxic effects in rats. No LD_50_ was reached at this dose, indicating that the LD_50_ exceeds 20.0 g/kg. This is more than 777 times the recommended daily intake for adults, indicating that *M. speciosa* has low acute toxicity [62]. According to the National Food Safety Standard [63] , the effects of the test substance on growth and development, hematology, blood biochemistry, and histopathology were comprehensively observed to characterize its subchronic toxicity. With *M. speciosa* root extract, it was shown that after 90 days of continuous oral administration to rats, no significant abnormalities were observed in body weight gain, blood biochemical parameters, or histopathological sections of multiple organs, indicating that the extract did not exhibit obvious subchronic toxicity at the experimental dose and has good food safety [64].

Single-cell gel electrophoresis is a classic method for detecting DNA damage in genetic toxicology. The results showed that the water extract of *M. speciosa* did not cause DNA damage in mouse cells from multiple organs within an appropriate concentration range, indicating an absence of genotoxicity [65]. In accordance with the aforementioned toxicological evaluation protocol, teratogenicity studies were conducted in pregnant rats. The results demonstrated that no maternal toxicity, embryotoxicity, or teratogenic effects were observed following the administration of the extract at various doses. Collectively, these findings confirm that the extract of *M. speciosa* does not exhibit developmental toxicity in pregnant animals at the tested doses, supporting its safety for consumption by pregnant women and the general population [66].

Heavy metals serve as critical safety indicators for plant-derived foods. In accordance with the *Green Industry Standards for the Import and Export of Medicinal Plants and Their Preparations * [67] , the maximum permissible limits for cadmium, arsenic, mercury, and lead in plant raw materials are 0.3 mg/kg, 2.0 mg/kg, 0.2 mg/kg, and 5.0 mg/kg, respectively. Analysis of the dried root product of *M. speciosa* revealed cadmium (0.06 mg/kg), arsenic (0.03 mg/kg), mercury (0.11 mg/kg), and lead (1.29 mg/kg) levels that were all below the established limits [8]. These findings indicate that the raw material poses no risk of heavy metal contamination, thereby further confirming its safety for use.

The health benefits of *M. speciosa* are supported by both traditional theory and modern clinical research. According to the ancient books such as *Sheng cao Yao xing Beiyao* and *Linnan Caiyaolu*, it is clearly stated that *M. speciosa* shows effects “regulating internal injuries, tonifying the kidney, and benefiting the health,” providing a theoretical basis for its use in treating conditions. In the folklore of the Hainan Li people, *M. speciosa* is a commonly used herb for postpartum conditioning, and it is often combined with *Artemisia indica*, *Leonurus japonicus*, and *Zingiber officinale* to promote postpartum recovery through immunomodulation [3]. These traditional uses demonstrate its potential as an immunomodulator.

In terms of anti-fatigue, the effect of *M. speciosa* corresponds to the traditional concepts of “tonifying deficiency” and “relaxing tendons and activating collaterals.” In clinical practice, Chinese medicine practitioners often combine *M. speciosa* roots with *Flemingia philippinensis* for the treatment of myasthenia gravis. For individuals engaged in high-intensity physical labor, athletes, and sub-healthy populations, the roots can be consumed daily as a decoction to relieve fatigue after physical exertion [27]. The dual status of *M. speciosa* as both a medicinal and an edible plant gives it unique advantages for daily use.

In addition, *M. speciosa* offers health benefits for respiratory, renal, and orthopedic disorders. According to the *Luchuan Bencao*, it “clears the lungs, relieves coughs, clears heat, and removes toxins” and is indicated for cough with blood, fever, thirst, and dysentery, making it especially suitable for individuals with respiratory sensitivities or mild inflammatory conditions. In patients with gouty arthritis, a clinical trial with 104 patients demonstrated that an herbal formula containing *M. speciosa* effectively relieved joint swelling and pain, improved disease-related weakness and fatigue, and helped restore daily mobility [68]. However, these findings are based on limited, small-sample clinical studies. The efficacy of these effects, appropriate dosages, and long-term applications still need to be validated through large-sample, multicenter clinical trials. Proprietary Chinese medicines containing *M. speciosa* roots, such as Zhuangyao Jianshen Tablets and Antirheumatic Liquid, can tonify the kidney and strengthen the bones, thereby regulating the function of internal organs and improving local circulation. Thus, they are suitable for kidney deficiency, orthopedic diseases, and lumbar and knee soreness caused by kidney diseases [69]. Collectively, the multifaceted evidence supports the multiple health benefits of *M. speciosa*.

## 7. Forms of Applications

**Figure 4:**
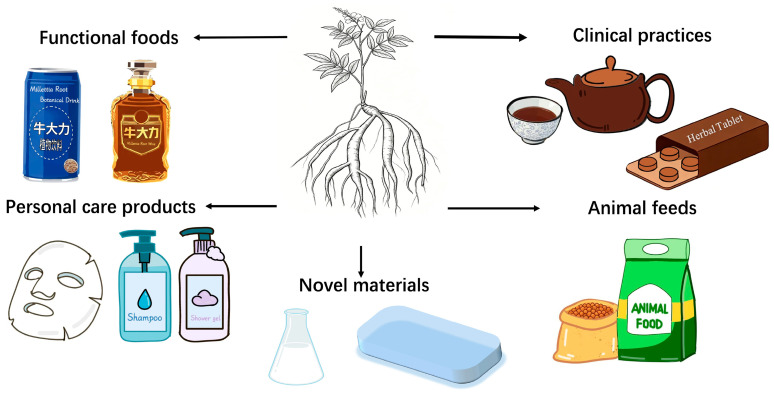
The applications of *M. speciosa* in multiple fields.

Due to the rich botanical resources and pharmacological effects of *M. speciosa*, its application has expanded from traditional uses to modern industries, and it has been developed into various products beyond its soup form (Figure 4).

As a medicinal and edible plant, *M. speciosa* not only exhibits clear pharmacological activities but also contains abundant nutritional components, conferring outstanding nutritional value. Furthermore, when boiled, it emits a delicate aroma, and its broth or tea has a pleasant aftertaste and appealing flavor, laying the foundation for its application in food products. Given these characteristics, together with the presence of various bioactive compounds in its extracts, the application of *M. speciosa* in the functional food sector is expanding, with its value particularly highlighted in immunity enhancement. To date, a range of products, including tea and beverages [70], have been developed.

For individuals with weakened immune systems, *M. speciosa* syrup can help alleviate suboptimal health conditions [71]. When blended with various herbs to make an instant tea, it provides the dual benefits of combating fatigue and boosting immunity [72], making it suitable for all ages. The wine-making process effectively extracts flavonoids [73], yielding a mellow-flavored *M. speciosa* wine that helps relax muscles and tendons while enhancing immunity. Furthermore, because *M. speciosa* roots are rich in starch, they can be directly processed into cell-broken powder for daily use as a health supplement or further developed into a variety of everyday foods, including noodles [74] and cookies [75]. However, it must be noted that existing research on *M. speciosa* in the food sector remains at a preliminary stage. To date, no systematic studies have been reported on key aspects such as the stability of active compounds during food processing, formulation optimization and product standardization, regulatory pathways for food safety, or the development of systematic commercial food applications. These gaps, to some extent, hinder the translation of *M. speciosa* from a raw material resource into industrial-scale food applications.

In the field of personal care, the application of *M. speciosa* is largely attributed to its anti-oxidant effects. Studies have demonstrated that *M. speciosa* extracts have excellent anti-oxidant activity and UV absorption capacity [76,77], which are attributed to the polysaccharides and flavonoids present in the plant. These properties provide a solid scientific basis for their use in skin care products. Based on these findings, researchers have successfully developed a series of products, such as facial masks, cleansers, sunscreens [78,79], shower gels, and shampoos [80,81], thereby maximizing the value of this plant’s resource utilization.

*M. speciosa* also has potential as a novel material. Due to its unique interfacial properties and anti-oxidant capacity, its polysaccharide component can serve as a natural emulsifier. It significantly improves the encapsulation efficiency, chemical stability, and oral bioaccessibility of unstable compounds such as β-carotene [82], suggesting its potential use in developing functional food delivery systems [83]. Acetylation of polysaccharides can further optimize their properties, and suitable raw materials can be selected during development based on specific needs. Due to its good 3D printing suitability, thermal stability, and anti-digestibility, *M. speciosa* starch can be used in both functional food and biomedical materials. In addition, the pH-responsive hydrogels constructed from its cellulose have good encapsulation efficiency and slow-release properties and are effective in improving the survival rate of probiotics in the gastrointestinal tract while maintaining their physiological functions [84]. These findings provide novel insights for the further development of *M. speciosa*. Beyond human applications, *M. speciosa* can also serve as a feed additive in aquaculture. The aqueous extract of *M. speciosa* effectively enhances intestinal immune function and disease resistance in fish, acting as a natural immunity booster [85] and demonstrating potential as an alternative to antibiotics. Additionally, *M. speciosa* polysaccharides improve the growth performance and meat quality of poultry, while also safeguarding animal health by enhancing immunity and anti-oxidant capacity, as well as regulating intestinal flora [86]. Collectively, these diverse applications comprehensively demonstrate the development potential of *M. speciosa*.

Despite extensive applied research, the industrialization of *M. speciosa* remains in its early stages, and most products currently available are raw or primarily processed goods, with marked limitations. First, product development based on identified active ingredients is highly inadequate. Current research has identified several active components in *M. speciosa*, such as polysaccharides and flavonoids. However, these components lack in-depth characterization and targeted utilization. Second, although its potential as a novel biomaterial has been experimentally verified, large-scale product development is yet to be achieved. This is mainly because research on the relevant properties of these components is still limited, making it difficult to guide practical applications. In addition, several technical bottlenecks remain in real-world applications, including constraints related to technology transfer and process optimization. Third, the industrial chain of *M. speciosa* remains immature. A complete industrial ecosystem has not yet been established. Poor coordination and inadequate supporting facilities across different stages of the industrial chain have become major obstacles to industrialization.

Future development should focus on creating products based on its active ingredients and expanding its applications in the health, novel biomaterial, and green agriculture sectors. It is also imperative to strengthen the integration of the industrial chain covering planting, active ingredient extraction, and product development to elevate the value of resource utilization.

## 8. Limitations of Current Research

Current research on *M. speciosa* still has limitations that hinder the transition from resource utilization to industrial translation. Regarding botanical resources, research on non-traditional plant parts such as the stems, leaves, and flowers remains insufficient. A comprehensive understanding of their tissue structures and chemical compositions is still lacking, which, to some extent, limits the full utilization of this species. Furthermore, research on the chemical composition of *M. speciosa* is still incomplete. The activities of alkaloids, triterpenoids, and sterols, in particular, have yet to be explored, and the pharmacodynamic basis of the plant requires further clarification. Existing pharmacological studies have been mostly confined to the animal and cellular levels, using crude extracts as the primary test substances. Experimental models and positive control drugs vary considerably across studies, and evaluation indicators have not been standardized. As a result, direct comparison of findings from different studies is difficult, and the specific mechanisms of action still require in-depth investigation. Although toxicological studies have systematically confirmed the safety of *M. speciosa* at the animal level, clinical safety evaluations remain notably lacking. Existing clinical research is limited to small-sample trials and lacks support from large-scale, multicenter studies. Consequently, the appropriate dosage and long-term efficacy of *M. speciosa* in humans have yet to be validated, which, to some extent, hinders its translational application. Regarding industrialization, *M. speciosa* remains at an early stage of development overall, with progress in the food sector lagging particularly behind. From a food science standpoint, there is a notable lack of research on the processing stability of bioactive compounds, formulation standardization, and regulatory frameworks for food safety. Moreover, most products currently on the market are limited to primary processing; the development of products derived from bioactive constituents remains inadequate, and the plants’ potential as a biomaterial has yet to be sufficiently exploited. In parallel, industrial chain coordination and supporting infrastructure are in pressing need of upgrading.

In summary, current research on *M. speciosa* exhibits limitations characterized by uneven resource utilization, unclear chemical composition and mechanisms, and weak clinical and translational foundations.

## 9. Conclusions and Perspectives

*M. speciosa* possesses significant comprehensive developmental value. Botanical studies have confirmed not only its wide resource distribution and stable supply, laying a foundation for sustainable industrialization, but also confirmed that the whole plant of *M. speciosa* has application value. Phytochemical studies have determined that *M. speciosa* contains a variety of chemical components and exhibits anti-oxidant, anti-inflammatory, and immune-enhancing, anti-fatigue, and other effects. This provides a scientific basis for its traditional efficacy as well as its health benefits to the human body. Toxicological studies have demonstrated its high safety, leading to its successful expansion from pharmaceuticals to food and personal care products, and even farming and new materials.

Considering current research and industry, several directions remain to be explored for the further development of *M. speciosa*. First, systematic research should be conducted on the chemical composition and biological activities of non-traditional parts such as stems, leaves, and flowers to discover new compounds, potential functions, and nutritional value. Meanwhile, further research on isolated alkaloids and triterpenoids should be conducted to further clarify the plant’s overall efficacy. Standardized indicators for each pharmacological activity should be established, and the underlying mechanisms require in-depth investigation. Subsequent efforts should include systematic toxicological evaluations to complete the safety profile, as well as clinical studies to provide human evidence supporting the pharmacological findings, thereby facilitating translational development.

For product development, fundamental research is required on the stability of *M. speciosa* during food processing, formulation standardization, and safety evaluation. A systematic technical framework and quality standards also need to be established. Concurrently, industrial chain coordination and supporting infrastructure must be strengthened to forge robust links among basic research, product development, and industrial application, thereby accelerating the systematic progression of *M. speciosa* product development.

To maximize resource value, *M. speciosa* should be developed in a part-specific manner. Its roots, rich in active components, are central to the great health industry, while its leaves, flowers, and fruits possess notable antibacterial potential for use in natural preservatives. Additionally, its abundant starch and cellulose provide raw materials for the production of novel biomaterials. At the same time, wild *M. speciosa* resources are under pressure from overharvesting, underscoring the need to strengthen conservation efforts. Existing cultivation techniques are relatively well-established and can, to some extent, ensure the sustainable use of these resources. Furthermore, a unified quality control system should be established to account for variations in production areas, harvesting periods, and cultivation methods. By identifying quality markers in conjunction with bioactive compounds, comprehensive quality evaluation standards can be developed to support industrial-scale production.

In summary, it is necessary to continue research on *M. speciosa* to fully explore its potential and provide solid evidence supporting its development and application.

## Data Availability

No new data were created or analyzed in this study.

## Abbreviations

The following abbreviations are used in this manuscript: MSP-1 Water-soluble polysaccharides from the roots of *Millettia speciosa* Champ. MSCP2 Polysaccharide fractions from the roots of *Millettia speciosa* Champ. NF-κB Nuclear factor kappa-B IL-1 Interleukin-1 IL-6 Interleukin-6 TNF-α Tumor necrosis factor-α TLR4 Toll-like receptor 4 MyD88 Myeloid differentiation primary response 88 BUN Urea nitrogen NIH mice NIH Swiss mice SD rat Sprague-Dawley rat KM mice Kunming mice ICR mice Institute of cancer research mice LPS Lipopolysaccharide RAW 264.7 cells RAW 264.7 macrophage cells CCl4 Carbon tetrachloride STZ Streptozotocin CUMS Chronic unpredictable mild stress SRBC Sheep red blood cell IgG Immunoglobulin G IgM Immunoglobulin M WBC White blood cell PGE2 Prostaglandin E_2_ LDH Lactic acid dehydrogenase LA Lactic acid CK Creatine kinase ·OH Hydroxyl radical DPPH· 1,1-diphenyl-2-picrylhydrazyl radical NF-κB p65 Nuclear factor-kappa B p65 subunit AST Aspartate aminotransferase ALT Alanine aminotransferase MDA Malondialdehyde α-SMA Alpha-smooth muscle actin Bax Bcl-2 associated x Collagen-1 Collagen type I LN Laminin HA Hyaluronic acid PCIII Procollagen III n-terminal peptide IV-C Type IV collagen TGF-β1 Transforming growth factor-β1 IL-1β Interleukin-1β IL-8 Interleukin-8 COX-2 Cyclooxygenase-2 TC Total cholesterol TG Triglyceride LDL-C Low-density lipoprotein cholesterol HDL-C High-density lipoprotein cholesterol IRS2 Insulin receptor substrate 2 PI3K Phosphatidylinositol 3-kinase Akt Protein kinase B GLUT4 Glucose transporter 4 UA Uric acid SCr  Serum creatinine XOD Xanthine oxidase IL-2 Interleukin-2 IL-4 Interleukin-4 IL-10 Interleukin-10 BDNF Brain-derived neurotrophic factor NE Norepinephrine 5-HT 5-Hydroxytryptamine Maoa Monoamine oxidase A Maob Monoamine oxidase B Ache Acetylcholinesterase Ido1 Indoleamine 2,3-dioxygenase 1 Comt Catechol-o-methyltransferase SOD Superoxide dismutase GSH-Px Glutathione peroxidase Sod1 Superoxide dismutase 1 Sod2 Superoxide dismutase 2 Sod3 Superoxide dismutase 3 Cat Catalase.
